# A Case of Isolated Celiac Artery Dissection Accompanied by Splenic Infarction Detected by Ultrasonography in the Emergency Department

**DOI:** 10.1155/2016/8608496

**Published:** 2016-04-11

**Authors:** Kazumasa Emori, Nobuhiro Takeuchi, Junichi Soneda

**Affiliations:** Department of Cardiovascular Surgery, Kobe Tokushukai Hospital, 1-3-10 Kamitakamaru, Tarumi-ku, Kobe-shi, Hyogo 655-0017, Japan

## Abstract

A 46-year-old male with a history of hypertension visited the emergency department (ED) by ambulance complaining of sudden pain in the left side of his back. Ultrasonography (USG) performed at ED revealed splenic infarction along with occlusion and dissection of the celiac and splenic arteries without abdominal artery dissection. Contrast enhanced computed tomography (CT) revealed the same result. Consequently, spontaneous isolated celiac artery dissection (SICAD) was diagnosed. Because his blood pressure was high (159/70 mmHg), antihypertensive medicine was administered (nicardipine and carvedilol). After his blood reached optimal levels (130/80 mmHg), symptoms disappeared. Follow-up USG and contrast enhanced CT performed 8 days and 4 months after onset revealed amelioration of splenic infarction and improvement of the narrowed artery. Here, we report a case of SICAD with splenic infarction presenting with severe left-sided back pain and discuss the relevance of USG in an emergency setting.

## 1. Introduction

Spontaneous isolated visceral artery dissection (SIVAD) is a sudden critical condition that may lead to aneurysmal formation, rupture, or arterial occlusion. SIVAD should be differentiated from visceral artery dissection (VAD) accompanied by aortic dissection. Recently, with the development of new imaging modalities, SIVAD can be diagnosed at an early stage and, if immediately treated, it can improve with conservative therapy. Here, we present a case of spontaneous isolated celiac artery dissection (SICAD) with splenic infarction presenting with severe left-sided back pain that was successfully diagnosed through ultrasonography at the emergency department.

## 2. Case

A 46-year-old male visited our emergency department complaining of sudden and severe left-sided back pain in the middle of July 2014. Costovertebral angle tenderness initially suggested a left-sided ureteral stone. His past medical history included hypertension and hyperlipidemia. He had no history of catheterization. Physical examination upon arrival revealed that his blood pressure was 159/70 mmHg, heart rate was 72 beats/min with regular rhythm, blood oxygen saturation was 99% under atmospheric conditions, and body temperature was 36.6°C. Blood chemistry analyses revealed a mildly elevated white blood cell count (10,500 cells/*μ*L), mildly elevated creatinine levels (1.07 mg/dL), and no abnormal coagulant dysfunction (80% prothrombin; 26.3 s activated partial thromboplastic time; 280 mg/dL fibrinogen; 94% haptoglobin; <5 *μ*g/mL fibrin/fibrinogen degradation products; D-dimer <0.5 *μ*g/mL).

**Figure 1:**
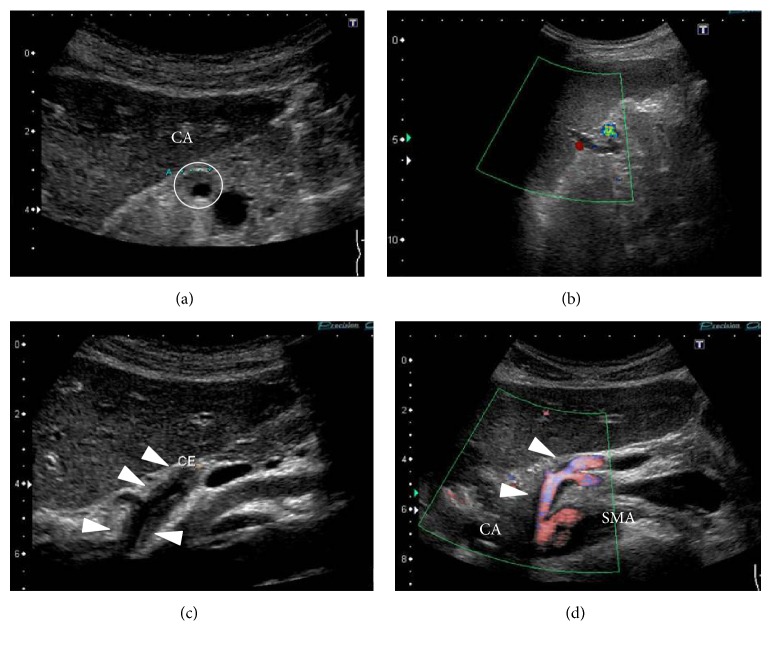
USG at arrival at ED. (a) Mural thrombus in the trunk of the celiac artery. (b) Splenic infarction is revealed by Doppler USG. ((c) and (d)) Mural thrombus extending from the trunk of the celiac artery to the distal splenic artery.

**Figure 2:**
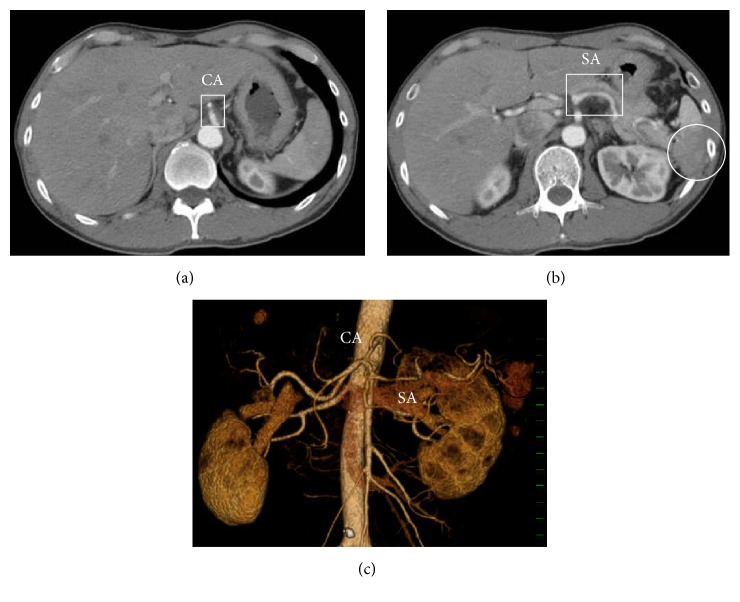
Contrast enhanced CT at arrival at ED. (a) Mural thrombus in the trunk of the celiac artery. (b) Splenic infarction and mural thrombus, expanding from the trunk of the celiac artery to the distal splenic artery. (c) Thrombosed artery extending from the trunk of the celiac artery to the distal splenic artery and splenic infarction shown using the volume rendering method.

**Figure 3:**
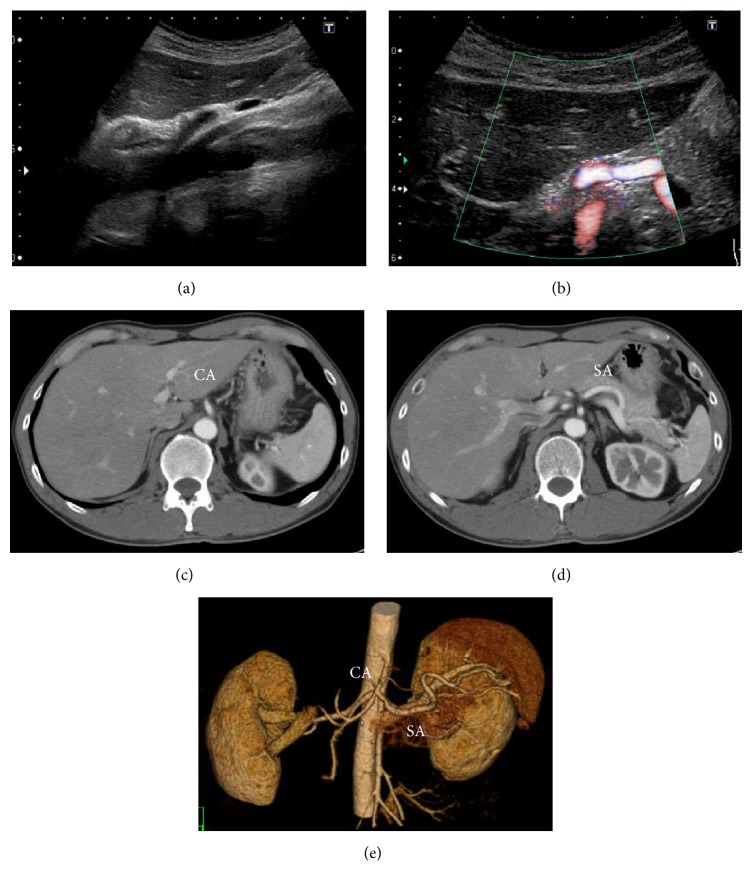
Follow-up USG and contrast enhanced CT. (a)–(d) USG and CT show improvement of the artery narrowed by thrombus.

Electrocardiography revealed a normal, regular heart rhythm without ST changes. Chest X-ray was normal without cardiomegaly or pleural effusion. Ultrasonography (USG) ruled out the presence of hydronephrosis or ureteral stones; however, Doppler USG revealed splenic infarction. Closer examination by USG revealed celiac artery dissection (CAD) with thrombotic occlusion of the false lumen extending from the trunk of celiac artery (CA) to the distal splenic artery (SA) (Figure 1). Consequently, CAD with splenic infarction was diagnosed. Contrast enhanced computed tomography (CT) revealed the same result; there was no finding of intestinal ischemia or aneurysmal formation, and blood flow in the dissected artery was preserved up to the distal SA (Figure 2). Conservative therapy was initiated with antihypertensive medicine (nicardipine and carvedilol) and rest. On day 8, follow-up USG and contrast enhanced CT revealed no deterioration of dissection or formation of arterial aneurysm and amelioration of splenic infarction. Conservative therapy was continued until he was uneventfully discharged on day 12. Ambulant follow-up was continued to control hypertension. USG and contrast enhanced CT performed 4 months after onset revealed amelioration of SICAD (Figure 3).

## 3. Discussion

VAD is usually accompanied by abdominal aortic dissection, and spontaneous visceral VAD not associated with aortic dissection is considered a rare condition. However, with the development of new imaging modalities, cases of VAD have increased. SICAD is a significant differential diagnosis of acute abdomen whose diagnosis is challenging. Bauersfeld [1] stipulated that the histological difference between SIVAD and aortic dissection is that SIVAD occurs between the intima and the external elastic layer, whereas aortic dissection occurs between the first and second part of the intima. SIVAD etiology is male dominant (4 : 1 ratio of males to females) usually occurring between 40 and 50 years of age (average: 56 years) [2]. Risk factors of SIVAD and CAD include atherosclerotic changes, hypertension, smoking, trauma, iatrogenic conditions, infections, pregnancy, and Marfan syndrome [3]. Another cause of CAD might include compression by the median arcuate ligament, in which there is continuous friction or stress on the celiac artery each time the diaphragm descends with respiration [4]. Our case was a relatively young male with a history of hypertension and an indistinct genetic background related to the condition. A main clinical symptom of SICAD is abdominal pain. If splenic infarction accompanies SICAD, clinical symptoms could include left-sided back pain and should be differentiated from ureter stones. If SICAD involves a branch of the hepatic artery, it may cause liver ischemia, leading to higher mortality. In such cases, surgical treatment or interventional stenting is recommended [3].

SICAD is sometimes accompanied by arterial aneurysm and clinical presentations include bleeding from ruptured aneurysm. Bret et al. [5] reported a case in which jaundice was caused by compression because of hepatic artery aneurysm and SICAD, which required percutaneous drainage and surgical resection of the aneurysm. Poor prognosis related to SICAD with aneurysm results from liver ischemia and bleeding of CA aneurysms and surgical treatment should be considered to treat these fatal conditions [5]. Although there was no evidence of aneurysm in our case, meticulous follow-up was needed to monitor the potential risks associated with aneurysm formation.

VAD is usually diagnosed through angiography, USG, CT, and/or magnetic resonance imaging (MRI)/MR angiogram (MRA). Until now, angiography has been considered the gold standard for diagnosing VAD; however, because of its invasiveness, it is being replaced by less invasive imaging modalities, such as USG, CT, and MRI/MRA [3]. With routine use of USG or CT in clinical settings to screen diseases, an increasing number of VAD without detectable symptoms have been diagnosed. It is also important to be aware of renal artery dissection in which patients present with back pain accompanied by renal infarction [6]. Whenever clinicians admit patients with back or flank pain, arterial diseases (e.g., splenic or renal infection) or SIVAD should be suspected and Doppler USG should be used.

SIVAD treatment includes conservative, antihypertensive, anticoagulant, and/or antiplatelet therapy, stent placement, bypass grafting, and resection of gangrenous intestines. Moreover, SIVAD treatment is classified into acute or chronic phase. Acute phase treatment includes emergency surgery if there are symptoms of aneurysmal rupture, impending rupture, or severe intestinal ischemia. It is unclear whether anticoagulant or antiplatelet agents effectively treat SIVAD; however, in some case reports, anticoagulant or antiplatelet agents were used for long-term management [7–9]. Conversely, there are some reports in which spontaneous isolated superior mesenteric artery dissection (SISMD) was successfully treated without anticoagulants [10, 11]. If decreased blood flow is caused by the development of SIVAD, thrombus formation is possible and can lead to organ ischemia. In some cases with reduced blood flow, anticoagulant or antiplatelet therapy may be helpful for preventing thrombus formation. In our case, anticoagulant or antiplatelet therapy was not provided regardless of whether splenic infarction resulted from SIVAD. However, splenic infarction was ameliorated by antihypertensive therapy. Thus, further research is required to determine which conditions require anticoagulant therapy. Yoon et al. [12] reported a case of SISMD that required stent replacement because of a progressively narrowed true lumen despite anticoagulant therapy. Aggressive therapy, including stent replacement or surgical treatment, is recommended in SIVAD cases, which fail to improve with conservative therapy. Even if conservative treatment is adopted in the first medical interview, meticulous follow-up is necessary because intervention is sometimes required to prevent further development of the condition. In the chronic phase, aneurysmal formation of over 20 mm or chronic intestinal ischemia, including abdominal pain after eating or weight loss, denotes candidates for surgical treatment. In our case, strict control of blood pressure without anticoagulant therapy was crucial for preventing recurrence for over 17 months after the initial presentation.

## 4. Conclusion

We reported a case of SICAD and splenic infarction presenting with severe left-sided back pain that was successfully diagnosed by USG. At ED, abdominal aneurysm, ureteral stone, and orthopedic diseases should be the differential diagnoses of back pain, and severe back pain caused by splenic infarction resulting from SICAD should be considered. USG may be a helpful imaging modality for properly diagnosing SICAD in emergency settings.
